# S110, a novel decitabine dinucleotide, increases fetal hemoglobin levels in baboons (*P. anubis*)

**DOI:** 10.1186/1479-5876-8-92

**Published:** 2010-10-08

**Authors:** Donald Lavelle, Yogen Saunthararajah, Kestis Vaitkus, Mahipal Singh, Virryan Banzon, Pasit Phiasivongsva, Sanjeev Redkar, Sarath Kanekal, David Bearss, Chongtie Shi, Roger Inloes, Joseph DeSimone

**Affiliations:** 1 Department of Medicine, University of Illinois at Chicago, 840 S. Wood St. Chicago, Illinois 60612-7323, USA; 2 Jesse Brown VA Medical Center, 820 S. Damen Ave., Chicago, Illinois 60612, USA; 3 Department of Hematologic and Blood Disorders, Cleveland Clinic, 9500 Euclid St., Cleveland, Ohio 44195, USA; 4 SuperGen, Inc., 4140 Dublin Blvd., Dublin, California 94568, USA; 5 Department of Animal Science/Molecular Biology, Agricultural Research Station, Fort Valley State University, Fort Valley, Georgia 31030-4313, USA

## Abstract

**Background:**

S110 is a novel dinucleoside analog that could have advantages over existing DNA methyltransferase (DNMT) inhibitors such as decitabine. A potential therapeutic role for S110 is to increase fetal hemoglobin (HbF) levels to treat β-hemoglobinopathies. In these experiments the effect of S110 on HbF levels in baboons and its ability to reduce DNA methylation of the γ-globin gene promoter in vivo were evaluated.

**Methods:**

The effect of S110 on HbF and γ-globin promoter DNA methylation was examined in cultured human erythroid progenitors and in vivo in the baboon pre-clinical model. S110 pharmacokinetics was also examined in the baboon model.

**Results:**

S110 increased HbF and reduced DNA methylation of the γ-globin promoter in human erythroid progenitors and in baboons when administered subcutaneously. Pharmacokinetic analysis was consistent with rapid conversion of S110 into the deoxycytosine analog decitabine that binds and depletes DNA.

**Conclusion:**

S110 is rapidly converted into decitabine, hypomethylates DNA, and induces HbF in cultured human erythroid progenitors and the baboon pre-clinical model.

## Background

**Figure 1:**
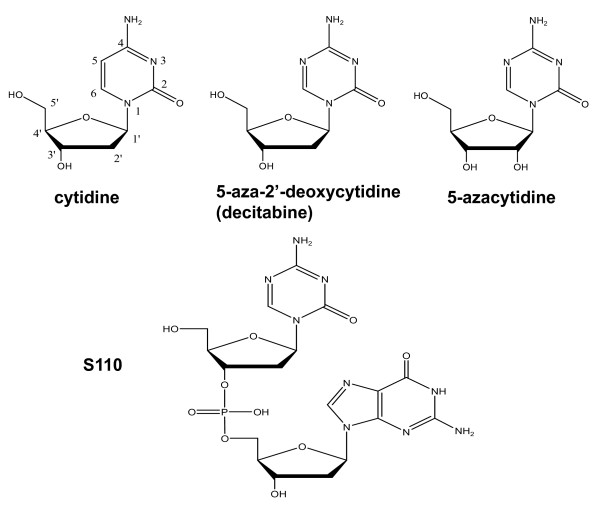
**Comparison of structures of cytidine, 5-aza-2-deoxycytidine, 5-azacytidine, and S110**.

Increased fetal hemoglobin levels are beneficial to patients with sickle cell disease and β-thalassemia. Patients with sickle cell disease with increased fetal hemoglobin levels have less pain crises [1] and longer life spans [2]. Therefore pharmacological agents that can elevate fetal hemoglobin have great potential as therapeutic agents. The DNA methyltransferase (DNMT) inhibitors 5-azacytidine and 5-aza-2'deoxycyidine (decitabine) have been shown to increase fetal hemoglobin levels in clinical trials in patients with sickle cell disease [3-6]. Although the clinical effectiveness of decitabine in alleviating the symptoms associated with the disease remains to be demonstrated in multi-center clinical trials, recent results in patients with severe sickle cell disease strongly suggest that this agent may have a major impact on the treatment of this disease [7]. Although decitabine and 5-azacytidine have a potential role as HbF inducers to treat β-hemoglobinopathies, these agents have pharmacological limitations including rapid destruction by the enzyme cytidine deaminase that is the principal barrier to oral administration [8,9]. The novel dinucleotide S110 (Figure 1) can also inhibit DNMT and is resistant to cytidine deaminase [10]. Hence, S110 could have advantages as a potential HbF inducer.

In this investigation our goal was to determine whether S110 increased fetal hemoglobin levels and reduced DNA methylation in cultured human erythroid progenitor cells and in baboons. Our results indicate that S110 administered by subcutaneous injection is rapidly converted to decitabine, hypomethylates the γ-globin gene promoter, and induces HbF. These results are the first demonstration that S110, a novel decitabine dinucleotide compound, can increase fetal hemoglobin and cause DNA hypomethylation in vivo and represent an important step towards understanding if S110 has a potential role in the treatment of β-hemoglobinopathies.

## Methods

### Drugs

Decitabine and S110 were obtained from SuperGen, Inc, Dublin, Ca.

### Cell Culture

Frozen CD34+ human cells purified from the peripheral blood of mobilized donors were purchased from Allcells, Inc. These cells were cultured in Iscove's media containing 20% fetal bovine serum, stem cell factor (SCF), erythropoietin (epo), estradiol, and dexamethasone [11]. On day 8, S110 or decitabine were added to the culture. After 24 hours, cells were transferred to fresh Iscove's media supplemented with 20% fetal bovine serum, epo, and insulin. One day 10, RNA was purified for analysis of globin mRNA expression. On day 11, lysates were prepared for high performance liquid chromatography (HPLC) analysis of globin chain expression and DNA was isolated for bisulfite sequence analysis.

### Baboon Treatments

Two baboons (*P. anubis*), PA 7256 and 7470, were used in these experiments. Prior to drug treatment, animals were phlebotomized to attain a hematocrit (Hct) of 20 by daily removal of 16-18% of the packed cell volume. Each animal was treated initially with S110 (1 mg/kg/d) for ten days, followed by a washout period prior to initiation of the second cycle of phlebotomy and subsequent administration of decitabine (0.5 mg/kg/d). The first dose of drug was administered IV followed by procurement of samples for pharmacokinetic analysis, with the remaining nine injections administered by subcutaneous injection on the subsequent days. Bone marrow (BM) aspirations from the hips were performed following the last day of drug administration. HbF levels were determined by alkali denaturation [12] and confirmed by HPLC [13]. All procedures were approved by Institutional Animal Care and Use Committee (IACUC) of the University of Illinois at Chicago.

### Real Time PCR Analysis of Globin mRNA

RNA was purified from cultured erythroid progenitors using the RNeasy Mini Kit (QIAGEN) according to manufacturer's instructions. RNA was treated with DNase I (Ambion) and used to prepare cDNA using kits (Fermentas). Levels of α-, γ- and β-globin transcripts were determined by real time PCR analysis using Taqman probe and primer sets (Applied Biosystems). Absolute numbers of α-, γ- and β-globin transcripts were determined by extrapolation from standard curves prepared from the cloned amplicons. Results were expressed as γ/γ + β mRNA ratio. Statistical significance was assessed using a two-tailed T test.

### HPLC analysis of Globin Chain Expression

For analysis of globin chain expression in cultured human erythroid progenitor cells, cells (5-10 × 10^6^) were harvested and washed three times in PBS. Lysates were prepared by addition of H_2_O to the packed cell pellet followed by three cycles of freezing and thawing in a dry-ice methanol bath. Analysis of globin chains was performed on a TSP Spectra HPLC system using a LiChristopher 100 RP-8 5 mM column and a gradient of acetonitrile-methanol-NaCl as described [13]. Absorbance was monitored at 215 nm. Quantitation of globin chains was performed by integration of peaks representing the separated α-, β-, and γ-globin chains using ChromQuest 4.1 software.

### Bisulfite Sequence Analysis

The DNA methylation status of 5 CpG sites (-54, -51, +5, +16, +48) within the 5' γ-globin promoter region was analyzed by bisulfite sequencing according to previously published methods [14,15]. Nucleated erythroid cells were purified from baboon bone marrow aspirates by Percoll density gradient sedimentation followed by immunomagnetic column (Miltenyi) purification using an anti-baboon red blood cell mouse monoclonal antibody (Clone E34-731, #551299, BD Bioscience) as the primary reagent and magnetically labeled rat anti-mouse IgG1 microbeads (Miltenyi) as the secondary reagent. DNA was isolated from purified baboon nucleated erythroid bone marrow cells and from cultured human erythroid progenitors using Qiagen blood mini kits. Bisulfite modification was performed as described following digestion with Hind III. The γ-globin gene promoter region was amplified by two rounds of PCR using semi-nested primers. The primer set BG1 (TATGGTGGGAGAAGAAATTAGTAAAGG) and BG2 (AATAACCTTATCCTCCTCTATAAAATAACC) were used in the first round and BG2 and BG5 (GGTTGGTTAGTTTTGTTTTGATTAATAG) in the second round. Amplicons were cloned in the PCR4 vector in the TOP10 E. coli strain. At least ten independent clones were sequenced from each sample.

### Pharmacokinetic Studies

Blood samples were collected from the femoral vein prior to drug administration (pre-dose) and 15, 30, 60, 120, 150, 180, and 240 minutes following intravenous administration of either decitabine or S110 in 3 mL K_2 _EDTA tubes pre-loaded with 8 μL of tetrahydrouridine (THU-500 μg/mL solution) and maintained on ice. Blood samples were centrifuged at 1,800 × g for 10 min at 4°C. The resulting plasma was decanted into a screw top tube and stored at -70°C until analyzed. Samples were shipped to SuperGen, Inc. on dry ice for analysis of decitabine and S110 levels. Levels of decitabine and S110 were determined using a liquid chromatography-tandem mass spectrometry method [16]. Values for HL LAMBDA (half life), Tmax (time of maximum concentration), Cmax (concentration at Tmax), AUCall (area under the curve from time of dosing to last observation), and AUCinf Obs (area under the curve from time of dosing to infinity) were calculated using WinNonLin version 5.0 (Pharsight).

## Results

### Effect of S110 in Human Erythroid Progenitor Cell Cultures

#### Globin Transcripts

**Figure 2:**
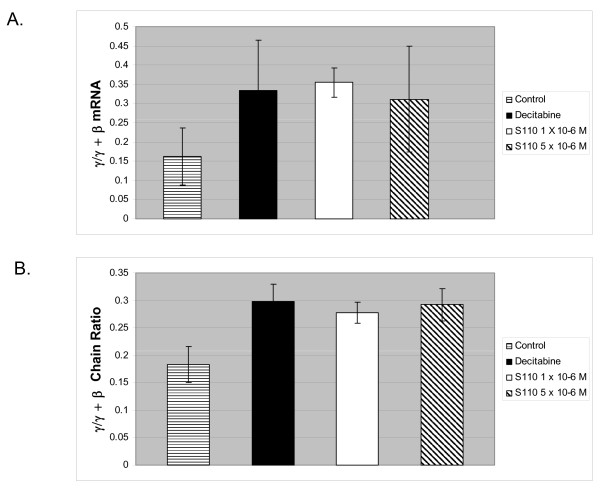
**Comparison of the effects of S110 and decityabine on globin gene expression in cultured human erythroid progenitor cells**. **A**. Effect of decitabine and S110 on expression of γ-globin mRNA in cultured human erythroid progenitor cells. Results are expressed as fold change (± SD) relative to untreated controls. The difference in γ/γ+β mRNA between the untreated controls and drug-treated cultures was significant (p < .05). **B**. Effect of decitabine and S110 on the γ/γ + β chain ratio in cultured human erythroid progenitor cells. The difference in γ/γ + β chain ratio between the untreated controls and drug-treated cultures was significant (p < .05).

Initial experiments were performed in human erythroid progenitor cell cultures to determine whether S110 increased γ-globin expression. Human CD34+ cells, purified from the peripheral blood of mobilized donors (AllCells), were cultured as described [11]. Because globin synthesis occurs between days 8 and 13 in these cultures [11], drugs, either S110 (1 or 5 μM) or decitabine (1 μM), were added on day 8. Analysis of levels of γ- and β-globin mRNA 48 hours post-decitabine addition showed that the γ/γ+β mRNA ratio in drug-treated cells was increased approximately twofold (p < .05) compared to untreated control cultures. (Table 1; Figure 2A). No significant difference in the α/γ+β mRNA ratio was observed between untreated controls and drug-treated cultures.

**Table 1:** Effect of S110 on γ-globin expression in human erythroid progenitor cell cultures.

Treatment	Dose (μM)	γ/γ + β mRNA	γ/γ + β polypeptide chain ratio
Control	0	0.162 ± .091 (n = 4)	18.3 ± 3.3 (n = 3)
Decitabine	1	0.337 ± .135 (n = 4)	29.8 ± 3.2 (n = 3)
S110	1	0.355 ± .038 (n = 4)	27.8 ± 1.9 (n = 3)
S110	5	0.310 ± .136 (n = 3)	29.2 ± 2.9 (n = 3)

The effect of decitabine and S110 on globin mRNA (n = 4) and globin chain expression (n = 3) was measured in cultured human erythroid progenitor cells. Difference in γ/γ+β mRNA and γ/γ+β chain ratios between untreated controls and drug-treated cultures was significant (p < .05)

#### Globin Chain Ratio

HPLC analysis of globin chain expression was also performed in human erythroid progenitor cultures treated with S110 or decitabine. Analysis of lysates prepared 72 hours following drug addition showed that the γ/γ+β chain ratio was increased 1.6 fold (p < .05) in cultures treated with decitabine and S110 compared to untreated controls. (Table 1; Figure 2B).

### DNA Methylation of the γ-globin Gene Promoter

**Figure 3:**
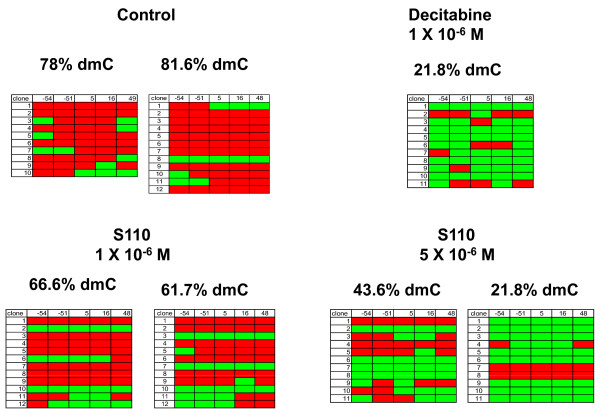
**Comparison of the effects of S110 and decitabine on DNA methylation of the γ-globin gene promoter region in cultured human erythroid progenitor cells**. The effects of decitabine and S110 on the DNA methylation of 5 CpG sites located within the 5' γ-globin promoter region are shown. Red rectangles = methylated CpG; green rectangles = unmethylated CpG. Results are expressed as the % deoxymethylcytosine (dmC) of cytosines located within CpG dinucleotides at positions -54, -51, +5, +16, and +48 with respect to the transcriptional start site of the human γ-globin gene promoter. Each row corresponds to the sequence analysis of an individual cloned PCR product derived from bisulfite-treated DNA. Results for each CpG site (-54, -51, +5, +16, +48) are in each corresponding column.

Bisulfite sequence analysis was performed to determine the effect of S110 on the level of DNA methylation of the γ-globin gene promoter. Marked DNA hypomethylation of the γ-globin promoter was apparent following treatment with either decitabine or S110 compared to untreated controls (Figure 3). The 1 × 10^-6 ^M decitabine dose and the 5 × 10^-6 ^M S110 dose induced similar levels of DNA hypomethylation

#### Effect of S110 in the Baboon

##### Fetal Hemoglobin

S110 was administered to baboons to evaluate its in vivo activity. Two phlebotomized baboons, PA 7256 and 7470, were treated with S110 (1.0 mg/kg/d) for ten days. The first injection was given IV and blood samples were obtained pharmacokinetic studies. The remaining nine drug treatments were administered by subcutaneous injection which avoids the need to anesthetize the baboons. An identical of course of decitabine using an equivalent molar dose (0.5 mg/kg/d), was given following a 60 day wash out period.

**Figure 4:**
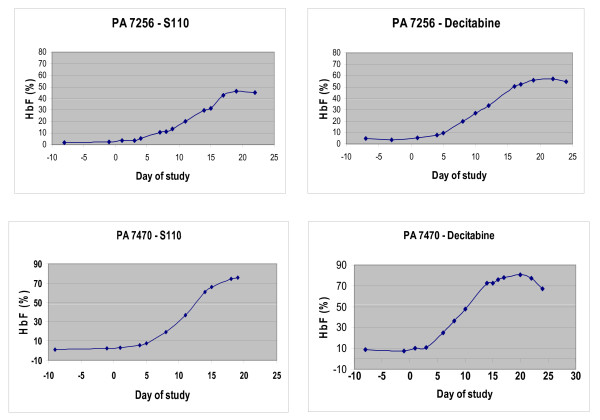
**Comparison of the effects of S110 and decitabine on fetal hemoglobin levels in baboons**. Kinetics of change in fetal hemoglobin levels during treatment with decitabine and S110 in PA 7256 and 7470. animals were treated with either S110 or decitabine between days 1-10.

Induction of HbF occurred following administration of both S110 and decitabine. Individual differences in maximal HbF attained were observed between the two baboons, and decitabine induced a slightly higher HbF response in each. The kinetics of response to S110 and decitabine were similar, with peak HbF attained approximately 10 days following the last day of drug administration (Figure 4).

##### DNA Methylation of the γ-globin Gene Promoter

**Figure 5:**
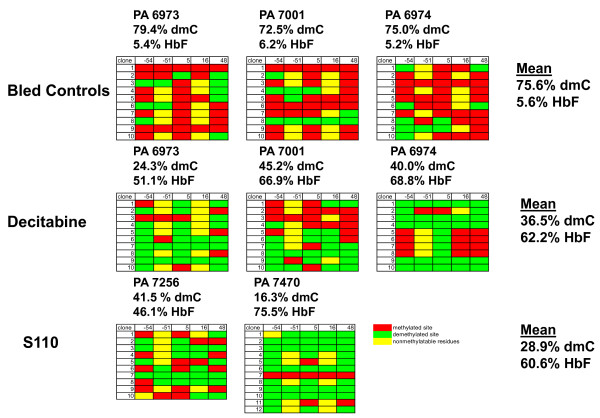
**Comparison of the effects of S110 and decitabine on DNA methylation of the γ-globin gene promoter region in baboons**. Red rectangles = methylated CpG; green rectangles = unmethylated CpG, yellow rectangles = polymorphic sites where no CpG dinucleotides are present. Results are expressed as the % deoxymethylcytosine (dmC) of cytosines located within CpG dinucleotides at positions -54, -51, +5, +16, and +48 with respect to the transcriptional start site of the baboon γ-globin gene promoter. Each row corresponds to the sequence analysis of an individual cloned PCR product derived from bisulfite-treated DNA. Results at each CpG site (-54, -51, +5, +16, +48) are within each corresponding column.

DNA was isolated from purified BM erythroid precursor cells obtained from baboons following the course of S110 administration to evaluate the effect of the drug on DNA methylation levels of the γ-globin gene promoter. The level of DNA methylation of 5 CpG sites within the γ-globin promoter was determined by bisulfite sequence analysis. S110 induced DNA hypomethylation of these CpG residues in both PA 7256 and 7470 compared to bled controls (Figure 5). The level of DNA hypomethylation of the γ-globin promoter induced by S110 was equivalent to that observed in three other baboons previously treated with decitabine [15].

##### Platelet and Neutrophils

**Figure 6:**
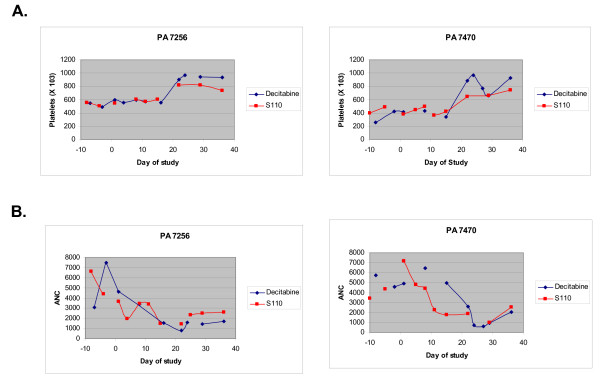
**Comparison of the effects of decitabine and S110 on platelets and Absolute Neutrophil Count (ANC) in baboons**. Platelet and absolute neutrophil count during the course of treatment of baboons with S110 and decitabine are shown. Animals were treated with either S110 or decitabine between days 1-10.

Both S110 and decitabine induced similar effects on neutrophil and platelet counts. Platelets counts rose approximately 2 weeks post-drug administration. The rise in platelet counts was mirrored by a decrease in neutrophils at this time following administration of both S110 and decitabine (Figure 6). This effect was previously observed in patients with sickle cell disease treated with decitabine [5].

##### Pharmacokinetic analysis

A summary of the pharmacokinetic data obtained is presented in Table 2. In baboons treated with S110, both S110 and decitabine were detected following administration of the drug. Peak levels of decitabine (17 ng/ml) were approximately 3 fold higher than peak levels of S110 (6 ng/ml) consistent with a rapid conversion of S110 into decitabine. Increased in vivo half life or AUC was not observed for S110 compared to decitabine when these drugs were administered intravenously.

**Table 2:** Pharmacokinetic data

Parameter	Units	Decitabine Injection (0.5 mg/kg)	S110 injection (1.0 mg/kg)	
Compound		Decitabine	S110	Decitabine
HL_LAMBDA_z	min	93	39	58
Tmax	min	30	16	15
Cmax	ng/ml	16	6	17
AUCall	min*ng/ml	1149	397	494
AUCINF_OBS	min*ng/ml	1463	516	593

Pharmacokinetic data calculated for baboons treated with decitabine and S110.

HLLambda z- half life, Tmax- time of maximal drug concentration, Cmax-concentration at Tmax, AUCall-area under the curve from time of dosing to last observation, AUCINF_OBS-area under the curve from time of dosing to infinity.

## Conclusion

Our results clearly demonstrate that subcutaneous administration of S110, a new decitabine dinucleotide, increases expression of γ-globin and reduces DNA methylation of the γ-globin promoter in cultured human erythroid progenitor cells, and also in baboons. The ability of S110 to induce HbF in vivo appears to be comparable to that of decitabine. Both decitabine and S110 are inhibitors of DNMT. The mechanism responsible for increased HbF by DNMT inhibitors is a matter of current controversy, however [17,18]. Decitabine has been observed to activate p38 MAP kinase and increase the rate of terminal erythroid differentiation in cultured erythroid progenitor cells [19], effects that have been associated with increased HbF [20,21]. Both S110 and decitabine decrease the level of DNA methylation of the γ-globin promoter, but the role of DNA hypomethylation in the mechanism of action of these drugs was not addressed in these experiments.

A previous report documented that S110 could demethylate and reactivate the expression of a silenced methylated p16INK4A tumor suppressor gene in cancer cell lines [10]. Results from these experiments strongly suggested that S110 dinucleotide was cleaved into individual nucleotides and nucleosides that were incorporated into DNA as the active form of the drug. It was speculated that S110 entered the cell as a dinucleotide where it was cleaved into its active form by phosphodiesterases. Our results demonstrate that S110 is rapidly cleaved in vivo into decitabine following intravenous administration. Pharmacokinetic analysis showed that levels of decitabine were approximately 3 fold higher than those of S110 following administration of S110. These results are consistent with rapid conversion of S110 into decitabine suggesting that S110 acts as a pro-drug. Similar molar doses of S110 and decitabine induce comparable levels of fetal hemoglobin, therefore most of the S110 must be bioavailable as the active decitabine. S110 is therefore an effective drug in vivo that produces effects comparable to decitabine when administered subcutaneously.

Effective oral administration of DNMT inhibitors requires either high doses of drug or co-administration of the cytidine deaminase inhibitor tetrahydouridine (THU; 8, 9). Even though S110 is resistant to cytidine deaminase, the rapid conversion of S110 into decitabine in serum suggests that S110 would not likely offer a significant advantage over decitabine for oral administration. To exploit the property of cytidine deaminase resistance to achieve effective oral delivery will require further modification of S110 to control its rapid conversion to decitabine.

## Abbreviations

HBF: (fetal hemoglobin); THU: (tetrahydrouridine); PBS: (phosphate buffered saline); HPLC: (high performance liquid chromatography); SCF: (stem cell factor); EPO: (erythropoietin); HCT: (hematocrit); IACUC: (Institutional Animal Care and Use Committee); DMC: (deoxymethylcytosine); ANC: (absolute neutrophil count); HLLAMBDA Z: (half life); TMAX: (time of maximal drug concentration); CMAX: (concentration at Tmax); AUCALL: (area under the curve from time of dosing to last observation); AUCINF_OBS: (area under the curve from time of dosing to infinity); BM: (bone marrow); DNMT: (DNA methyltransferase)

## Competing interests

DL, YS, KV, MS, and VB, and JDS have no competing interests. These investigators were not employed by SuperGen and received no funds from SuperGen for this work. SuperGen supplied S110 and conducted pharmacokinetic studies but supplied no additional funds to the University of Illinois at Chicago, Jesse Brown VA Medical Center, or its employees to conduct these studies. PP, SR, SK, DB, CS, and RI were employees of SuperGen, Inc.

## Authors' contributions

DL, KV, MS, and VB performed the experiments in human erythroid progenitor cells and baboons. PP, SR, SK, and DB developed the S110 reagent.

CS, and RI performed the pharmacokinetic analysis. DL, YS, and JD interpreted the data and wrote the manuscript. All authors read and approved the final manuscript.
